# Identifying peaks in *-seq data using shape information

**DOI:** 10.1186/s12859-016-1042-5

**Published:** 2016-06-06

**Authors:** Francesco Strino, Michael Lappe

**Affiliations:** Qiagen Aarhus, Silkeborgvej 2, Aarhus, 8000 DK Denmark

**Keywords:** Peak calling, ChIP-seq, DNase-seq, Hotelling observer, CLC shape-based peak caller

## Abstract

**Background:**

Peak calling is a fundamental step in the analysis of data generated by ChIP-seq or similar techniques to acquire epigenetics information. Current peak callers are often hard to parameterise and may therefore be difficult to use for non-bioinformaticians. In this paper, we present the ChIP-seq analysis tool available in CLC Genomics Workbench and CLC Genomics Server (version 7.5 and up), a user-friendly peak-caller designed to be not specific to a particular *-seq protocol.

**Results:**

We illustrate the advantages of a shape-based approach and describe the algorithmic principles underlying the implementation. Thanks to the generality of the idea and the fact the algorithm is able to learn the peak shape from the data, the implementation requires only minimal user input, while still being applicable to a range of *-seq protocols. Using independently validated benchmark datasets, we compare our implementation to other state-of-the-art algorithms explicitly designed to analyse ChIP-seq data and provide an evaluation in terms of receiver-operator characteristic (ROC) plots. In order to show the applicability of the method to similar *-seq protocols, we also investigate algorithmic performances on DNase-seq data.

**Conclusions:**

The results show that CLC shape-based peak caller ranks well among popular state-of-the-art peak callers while providing flexibility and ease-of-use.

## Background

In order to identify functional elements in a genome, a number of experimental high-throughput techniques have been developed for investigating specific interactions between proteins and DNA. These protocols provide us with a deeper understanding of gene-regulatory and epigenetic mechanisms by identifying, for example, Transcription-Factor Binding Sites (TFBS), open chromatin regions or the location of epigenetic marks.

In broad terms, these techniques chemically cross-link proteins to those stretches of DNA they are bound to in vivo. After shearing the DNA, a protein of interest is extracted along with the cross-linked DNA fragments from the cell-lysate using specific antibodies. Following this *Ch*romatin *I*mmuno-*P*recipitation (ChIP) step, the short stretches of DNA attached to the protein of interest are identified by high-throughput *seq*uencing.

For any targeted protein and a given cell-line or condition, this results in several million reads of raw sequencing data. Usually a control experiment is performed where the immuno-precipitation step is left out or an antibody that is not specifically binding to the target genome is used. For example, the ENCODE Project (*ENC*yclopedia *O*f *D*NA *E*lements) has produced data on hundreds of regulatory factors (see http://encodeproject.org/) in mouse and human. For more in-depth information we recommend the “ChIP-seq guidelines and practices of the ENCODE and modENCODE consortia” [1, 2].

Furthermore, there are already numerous experimental protocols related to ChIP-seq available and new protocols are published all the time. To name a few prominent examples, ChIP-exo [3] is a derivative of ChIP-seq where exonucleases are used to identify the genomic location of DNA-protein binding-sites with higher resolution. DNase-seq (DNase I hypersensitive site sequencing [4, 5]), ATAC-seq Assay for Transposase-Accessible Chromatin with highthroughput sequencing [6]) and FAIRE-seq (Formaldehyde-Assisted Isolation of Regulatory Elements sequencing [7]) are used to identify accessible regions in the genome, and MNase-seq (Micrococcal Nuclease sequencing [8]) is used to identify nucleosome positioning. Although each experimental technique uses different procedures for fragmentation and enrichment [9], the computational processing in terms of mapping the sequencing data and analysing the resulting signal in genomic context is similar to processing ChIP-seq data. Hence it is unsurprsing to see pipelines developed for ChIP-seq analysis routinely being applied to data produced with other protocols. Therefore, we initially discuss the analysis of ChIP-seq data and later investigate how algorithms developed for ChIP-seq perform on DNase-seq data.

ChIP-seq experiments are also increasingly used to investigate histone modifications. In contrast to transcription factors, most histone marks are of variable length and can span across entire gene bodies. Although the experimental procedures are similar, the resulting data needs to be treated accordingly. Some existing approaches such as HOMER stitch together narrow peaks to avoid the computational cost of finding regions of variable length. Since histone marks tend to be associated with genes, we opted for the use of existing annotations to classify genes according to peak shape as a practical trade-off between computational complexity and biological sensitivity.

### State of the art

The initial step for all downstream computational analyses of ChIP-seq data starts by mapping the reads to a reference genome. Obviously, the quality of the read mapping has an impact on the downstream analysis results. However, details of the mapping process are beyond the scope of this paper and we will assume that an accurate read mapping is provided. For the performance comparison, all peak callers used the same read mappings as input.

**Fig. 1:**
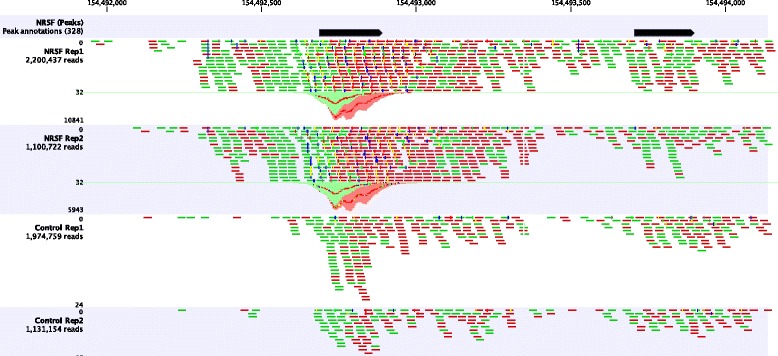
Coverage graphs and mapped reads from the NRSF dataset, showing a region of human Chromosome 1. The uppermost track marks two regions classified as positive peaks in the reference dataset with *blue arrows*. The two tracks below visualise the read mapping of the two replicate ChIP-samples. The two lowermost tracks display the reads from the two control samples. In all tracks, forward reads are shown in *green* and reverse reads are shown in *red*. Below the reads of the ChIP-seq sample data at the peak regions, the coverage graph of forward and reverse reads is shown

By plotting the number of reads mapped to genomic coordinates as a so-called coverage graph, consistent and specific binding sites of the protein of interest become visually apparent as peaks (see Fig. 1). Rather than identifying such regions by eye, subsequent bioinformatics analysis aims at the reliable automated identification of protein-DNA binding events from the read mapping - a process referred to as peak calling.

Many different approaches to peak calling have been developed. The density of the ChIP signal can be analysed directly (Findpeaks [10]) or compared to a control signal (CCAT [11], CisGenome [12], Erange [13], PeakSeq [14]). Signal processing approaches including Gaussian Kernel Density Estimation (FSeq [15], QuEST [16]), Hotelling filters (DFilter [17]) and wavelets [18] have also been applied to this kind of signal. Many statistical approaches such as Poisson distributions (SISSRS [19], HOMER [20], MACS [21]), negative binomial distributions (ZINBA [22]), rank-statistics (W-ChIPeaks [23]), and Cramér-von Mises test (Qeseq [24]) have been used. Other approaches include clustering approach (SICER [25]), Hidden Markov Models (ChIPDiff [26], RSEG [27]), tree shape statistics (TPIC [28]), shape recognition (Triform [29]) and probabilistic models (SignalSpider [30]).

From a computational viewpoint, the main lessons learned from the current generation of peak calling algorithms can be summarised as follows: The success of peak calling depends on how well the statistical model of the input signal can be fitted to the data under consideration. In this context, parameterising a peak caller can be seen as tweaking its intrinsic model to improve the fit to the data. However, this requires in-depth knowledge of the underlying algorithm and statistical model and a good grasp of how the behaviour is affected by the parameters. Therefore the fine-tuning of parameters remains a “black art” to most biologists who want to analyse the results of their ChIP-seq experiments. Since parameter optimisation is a hard and time-consuming task, it is recommended [1] to focus on improvements in experimental design in order to obtain better input data rather than attempting to optimise the downstream bioinformatics pipeline.

In recent literature, it has been observed that current peak callers may miss peaks that are clearly visible to the human eye. More precisely the peaks exhibit distinct shapes which act as visual cues. However, as stated by Rye et al. [31], “peak shape information is not fully exploited in the evaluated programs”. The gap between peaks apparent to expert users and what is recognised algorithmically has been observed time and again in recent literature [32].

One approach to improve peak calling is to take more of the observed specific characteristics of existing ChIP-seq datasets into account and encode them directly into the algorithm (see e.g. [29]). However, hard-coding more specific models into the heuristic is not a sustainable path for future developments in the long run, as this procedure may be overfitting certain kinds of datasets. Specially given the increasing variety of ChIP-seq related experimental protocols, this approach would ultimately lead to a similar variety of specialised heuristics. This variety and specialisation will make it increasingly difficult to update, test, and release algorithms while leaving users confused as to which tool and version is optimally suited to their data at hand.

Nevertheless, novel peak-shape recognition algorithms have been shown to outperform existing heuristics, identifying peaks that were missed previously [17, 33]. Generally speaking, in contrast to a hard-coded internal model these approaches “learn” the peak-shape from the underlying data. In the “learning-phase” an initial set of positive examples is identified, i.e. regions that unambiguously contain peaks. This phase is shared with other methodologies such as MACS and DFilter. From this initial set, a computational representation of the specific peak-shape is constructed. This representation is then applied to the entire dataset to perform the automated peak calling, based on a suitable statistical framework.

### Aims for a next-generation peak caller

Reflecting on the recent developments in the field combined with the practical lessons learned from a number of different current peak calling algorithms and the potential advances offered by the aforementioned shape-based approaches, we formulate the main requirements for a new peak calling toolset as follows:

**Generality** In order to keep up with the steadily rising number of experimental protocols that require peak calling for data-analysis, the algorithmic engine has to be general enough to be applicable across datasets from many different *-seq technologies (i.e. ChIP-seq, DNase-seq, etc.). The toolset should be swiftly adaptable to new datasets as they become available without expensive recoding efforts.

**Specificity** For achieving optimal results, the specific characteristics of the peaks need to be recognised by the algorithm. The optimisation and parameterisation for the task at hand should not sacrifice the generality of the underlying algorithmic implementation.

**Robustness** Rather than inventing ad-hoc scoring schemes, the algorithms need to be built on a mathematically and statistically well-founded framework. In particular, we employ methodologies from digital signal processing and machine learning, which have been extensively studied and are deeply understood.

**Simplicity** Despite the algorithmic and statistical complexity of the data-analysis task, the implementation needs to be suitable for a general audience. This translates into minimising or even eliminating the need for parameterisation and automating the most common tasks. At the same time, the algorithm needs to be transparent about its results and intuitive to use, such that advanced users can adopt the tools easily to their needs.

### New approach

Some of the aims formulated above may seem to be contradictory or even mutually exclusive to each other at first sight. However, peak calling constitutes a special case of signal detection algorithms that “can be solved by adapting ‘uniform’ and ‘formally optimal’ techniques from the signal processing literature” [17]. In addition to being general such that signals of arbitrary shape can be processed, it is based upon a well studied framework from signal processing theory. The specific shape of the signal is learned from a number of “positive” and “negative” regions (or noise) where the signal is absent or is the result of a sequencing artefact. The resulting shape of the signal minus the noise is encoded in a vector (the so-called Hotelling-observer, named after the mathematical statistician Harold Hotelling [34]), which is then evaluated against the data-stream. This resulting filter contains the information needed for peak detection and can easily be transferred and applied to other datasets as well. This approach lends itself to visual parameterisation by example such that advanced users could define the set of regions from which the filter is constructed, making it transparent as to what pattern the algorithm is detecting. Examples of approaches along this rationale are described in [17, 29, 35]. At the same time, the underlying implementation remains independent of the peak-shape it is detecting - analogous to text-search algorithms being independent of the text pattern or regular expressions.

In order to build specific peak-shape filters without extensive manual annotation of positive and negative regions, we take into account that the vast majority of peak callers hardly disagree about top-scoring peaks [36]. The differences in performance become apparent only for less obvious peaks; it is in this “grey zone” where the fit between the data and the intrinsic statistical model of the algorithms decides about their relative performance. This observation suggests a strategy for boot-strapping the shape-based approach: There are sufficient positive regions that can be safely and unambiguously identified by any of the currently available methods in a first pass. A more specific model of the peak-shape is then inferred from these clear-cut examples, allowing the algorithm to tune itself automatically to the data at hand. A second peak-detection step is performed, resulting in a much more sensitive peak-detection overall.

## Results and discussion

### Signal detection using a Hotelling-filter

**Fig. 2:**
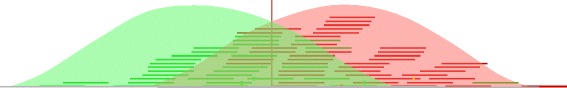
Distribution of forward (*green*) and reverse (*red*) reads around a binding site of the transcription factor NRSF. The centre of the putative binding site is indicated by a *red vertical line*

Since the shape of the signal from ChIP-seq data depends on which protein was targeted in the immuno-preciptation reaction [17, 35], the CLC shape-based peak caller is designed to take the characteristic peak-shape into account. For example, the typical signal shape of a transcription factor binding site like NRSF shows a high concentration of forward reads followed by a high concentration of reverse reads (Fig. 2).

**Fig. 3:**
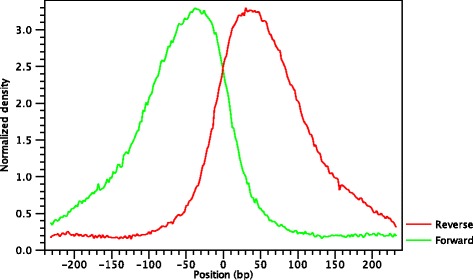
Average peak shape of the transcription factor NRSF

The average shape of the positive regions of the NRSF transcription factor for the forward and reverse strands is shown in Fig. 3.

The CLC shape-based peak caller makes use of both the characteristic peak shape and enriched read coverage to identify peaks in *-seq data. Next, we will outline the individual steps of the entire ChIP-seq peak calling pipeline, starting from quality control and normalisation of the data, describing the fundamentals of using a filter, learning a characteristic peak shape from highly enriched regions and calling peak regions including boundary refinement. Finally, we describe how these steps are working together to result in a highly automated peak detection pipeline that provides near optimal results without the need for extensive parameterisation by the user.

#### Quality control of ChIP-seq data

**Fig. 4:**
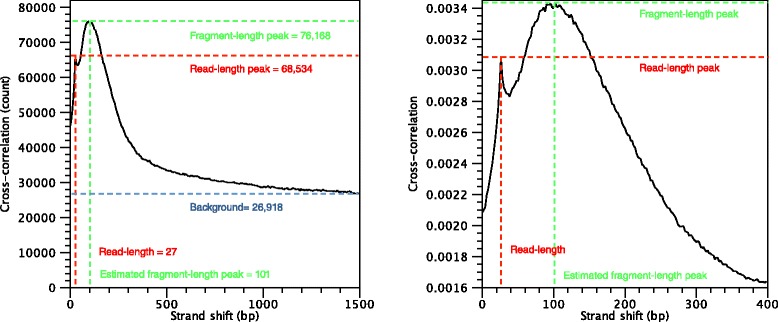
Inspection of the cross-correlation plot of a ChIP-seq experiment. On the *left*, full correlation profile. On the *right*, blow-up of the interesting region between 0 and 400 bp. Note that in the *right* plot, the cross-correlation values have been normalised so that the area under the *curve* is 1

**Fig. 5:**
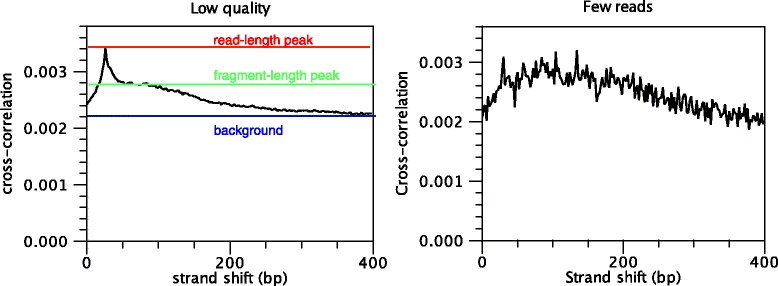
Cross-correlation plots of two low-quality ChIP-seq datasets. On the *left*, the read-length peak is significantly higher than the fragment-length peak (relative strand correlation of around 0.5), indicating potential problems in the immune-precipitation step. On the *right*, a very noisy cross-correlation profile indicates a ChIP-seq experiment where a very small number of reads was sequenced

During the first step of the analysis, the CLC shape-based peak caller computes several quality measures to check whether the input data satisfy the assumptions made by the algorithm. These measures can be derived from the cross-correlation profile between reads mapping to the forward and to the reverse strand. This plot is often used to investigate the quality of ChIP-seq experiments [1, 2]. A correlation profile is shown in Fig. 4. Those quality measures have been investigated by the modENCODE consortium and are described in more detail in [1]. The cross-correlation profile shown in Fig. 4 is typical of a successful ChIP-seq experiment. On the other hand, cross-correlation plots without a pronounced fragment-length peak are typically reflective of poor quality and ragged cross-correlation profiles are typically caused by low yield (Fig. 5).

The cross-correlation function typically has a maximum when the value of the strand-shift is close to the length of the DNA fragment being sequenced. This is indicated in green in Fig. 4. This peak is a characteristic feature of a ChIP-seq experiment. One can expect a pronounced peak around the fragment length because the frame shift between reads mapping to the forward and to the reverse strand near a typical transcription factor binding site (Fig. 2) is on average equal to the fragment length [1, 35, 37]. Therefore, the (relative) height of this peak can be considered a proxy for the quality of the ChIP-seq experiment. Finally, the location of this peak can be used to estimate the average length of the DNA fragments after the fragmentation step (e.g. sonication or MNase digestion).

#### Normalisation

The CLC shape-based peak caller analyses the genomic coverage of the reads. For each read mapping, the 5’ position of the reads mapping to the forward strand and the 3’ position of the reads mapping to the reverse strand are extracted. For each genomic position *x*, we define *f*(*x*) as the number of reads mapping to the forward strand where *x* is the 5’ position and *r*(*x*) as the number of reads mapping to the reverse strand where *x* is the 3’ position. A quantile standardisation is then applied to *f*(*x*) and *r*(*x*) such that the normalised coverage functions *f*^′^ and *r*^′^ follow a standard normal distribution, i.e. $f'(x), r'(x) \sim \mathcal {N}(0,1)$.

### Discovering obvious peaks

**Fig. 6:**
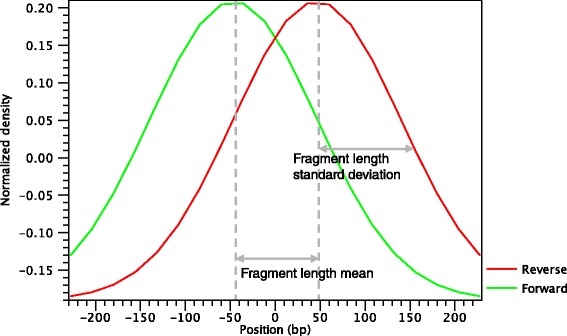
A Gaussian filter for the transcription factor NRSF

The next step of the CLC shape-based peak caller is to build a filter, which can be used to identify genomic regions whose read coverage profile matches the characteristic peak shape and to determine the statistical significance of this match. In order to build such a filter, examples of positive (e.g. ChIP-seq peaks) and negative (e.g. background noise, PCR artefacts) profiles are needed as input. The rationale is that regions with very high coverage in the ChIP-seq experiment are positive examples and regions with high coverage in the control and low in the experimental ChIP-seq data are negative examples, as they most likely originate from regions with strong sequencing biases or from PCR artefacts. Positive regions are generally easy to find and are typically found by every peak caller [36]. The CLC shape-based peak caller finds these peaks by building a Gaussian filter based on the mean and variance of the fragment length distribution, which are inferred from the cross-correlation profile (Fig. 5). An example of a filter is shown in Fig. 6.

**Fig. 7:**
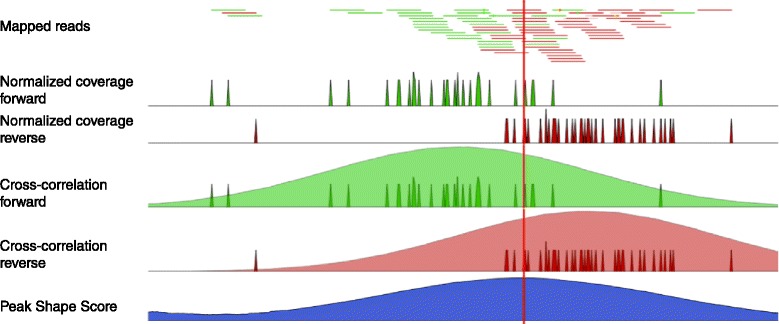
Application of a Gaussian filter (Fig. 6) to ChIP-seq data. In the first step, the normalised coverage for reads mapping to the forward (*green*) and reverse (*red*) strand are computed. Later, for a genomic position, the cross-correlation between the forward coverage and the filter shape centred at that position is computed. An analogous procedure is performed for reads mapping to the reverse strand. Their sum constitutes the Peak Shape Score (*blue*)

The filter is then applied to the input data as shown in Fig. 7 and the result is a score that indicates how likely a genomic position is to be a centre of a peak. In detail, the score is calculated as

(1) $$  \text{score} = \textrm{genomic coverage} \star \text{filter},  $$

where ⋆ denotes the cross-correlation operator. The cross-correlation between a function and a filter can be described as follows: For each genomic position *x*, we extract the genomic coverage profile of a window centred at *x*. We multiply this profile by the peak shape filter and we sum the result. The resulting number indicates how well the shape of the filter is matched. The score will reach a maximum at the centre of a peak. Peaks are then identified as the regions whose centres are the genomic positions with highest score and whose size is the size of the filter.

Similarly, the set of negative examples is identified by running a Gaussian filter. If control data is available, the negative examples are identified as regions where the genomic coverage in the control dataset is higher than the one in the ChIP dataset. However, if there is no information to build a negative profile from, the negative profile is estimated from the sequencing noise.

#### Learning the peak shape

**Fig. 8:**
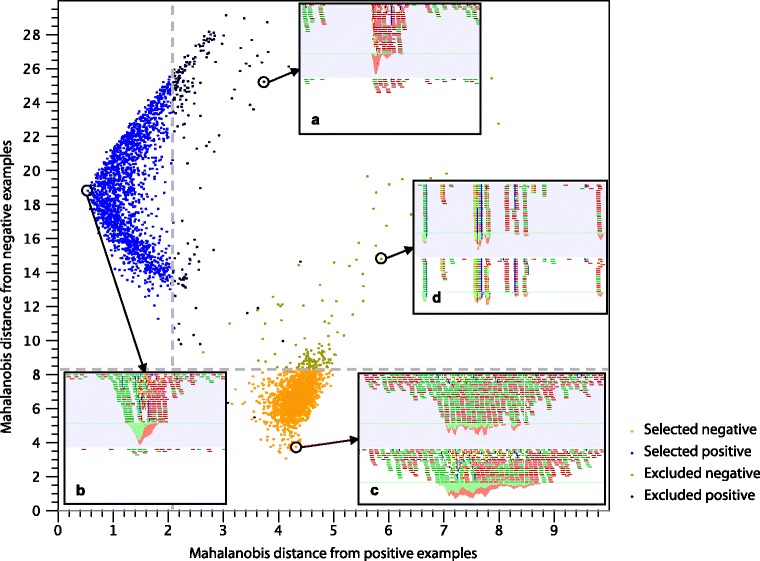
Outlier detection. The plot shows the Mahalanobis distance of each candidate region to the set of positive and negative profiles. The threshold values for positive and negative samples are indicated by a vertical and a horizontal line, respectively. Inner figures **a**, **b**, **c**, and **d** show examples of profiles, displaying reads matching to ChIP-seq data (*top part, light blue background*) and reads matching to the genomic control (*bottom part, white background*): **a** A noisy outlier is excluded from the set of positive profiles; **b** A very clear peak is classified as positive profile; **c** A peak with a high coverage in the control is classified as negative profile; **d** A region with abnormally high coverage is excluded from the set of negative profiles

After identification of positives and negatives, outliers are removed. The Mahalanobis distance [38] between each example and the group of positives and negatives is computed and candidate regions with highest Mahalanobis distance are removed (see Fig. 8). The Mahalanobis distance was chosen as metric because it gives more importance to the most conserved part of the filter (typically, the maxima) than to the less conserved (typically, the noisy edges) and corrects for correlation between genomic positions.

The threshold is chosen using a robust estimator and a confidence level of *α*=0.95 as

(2) $$ \text{threshold} = \text{median}(d_{i}) + \Phi^{-1}(\alpha) \frac{\text{m.a.d.} (d_{i})}{\Phi^{-1}(0.75)},  $$

where *d*_*i*_ is the Mahalanobis distance between the region *i* and its reference group, *Φ*^−1^ indicates the quantile of the standard normal distribution, m.a.d. indicates the median absolute deviation, and the term $\frac {\text {m.a.d.} (d_{i})}{\Phi ^{-1}(0.75)}$ is a robust estimate of the standard deviation under the assumption of normal distribution [39].

Once the positive and negative regions have been identified, the CLC shape-based peak caller learns a filter that matches the average peak shape, which we term Peak Shape Filter. The filter implemented is called Hotelling Observer [34] and was chosen because it is the matched filter that maximises the Area Under the Curve of the Receiver Operator Characteristic (AUC-ROC), one of the most widely used measures for algorithmic performance.

**Fig. 9:**
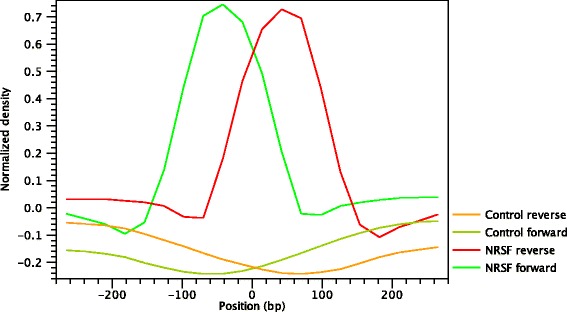
Peak Shape Filter for the transcription factor NRSF

The Hotelling observer *h* is defined as:

(3) $$  h = \left(\frac{R_{p} + R_{n}}{2}\right)^{-1} \left(\mathrm{E}\left[X_{p}\right] - \mathrm{E}\left[X_{n}\right]\right),  $$

where E[ *X*_*p*_] is the average profile of the *positive* regions, E[ *X*_*n*_] is the average profile of the *negative* regions, while *R*_*p*_ and *R*_*n*_ denote the covariance matrices between the *positive* and *negative* profiles, respectively. The Hotelling Observer has already previously been successfully used for calling ChIP-seq peaks [17]. An example of Hotelling observer is shown in Fig. 9.

Even though the shape of the Hotelling Observer is typically similar to the average profiles (Fig. 3), it is in fact modelling the shape that is maximally discriminative between positive and negative example and is therefore more similar to the difference between positive and negative examples.

#### Peak shape score

The Peak Shape Filter is applied to the experimental data and a score is calculated at each genomic position (Fig. 7). The score is obtained by extracting the genomic coverage profile of a window centred at the genomic position and then comparing this profile to the Peak Shape Filter. The result of this comparison defines the Peak Shape Score. The Peak Shape Score is standardised and follows a standard normal distribution, so a *p*-value for each genomic position can be calculated as *p*-value=*Φ*(−Peak Shape Score of the peak centre), where *Φ* is the standard normal cumulative distribution function.

#### Peak-detection

**Fig. 10:**
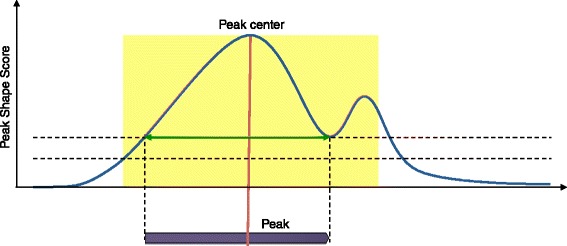
Peak calling. After the centre of the peak is identified (*red line*), the values in a window around the centre are analysed (*yellow*). The minima on the left and right side are identified (*dashed horizontal lines*) and the higher value is used as threshold. Next, the peak is expanded (*green arrows*) by adding all genomic position whose value is higher than the threshold to the *left* and to the *right* side of the centre. The final peak is shown in *purple*

Finally, peaks are called by first identifying the genomic positions whose *p*-value is higher than the specified threshold and which do not have any higher value in a window around them. The size of this window is determined by the filter as the longest distance between two positive values in the filter. These maxima define the centre of the peak, while the peak boundaries are identified by expanding from the centre both left and right until either the score becomes 0 or the peak touches a window boundary (Fig. 10).

#### The CLC shape-based peak caller

The CLC shape-based peak caller implements all the steps previously described in a single and easy-to-use algorithm. It is available as part of the CLC Genomics Workbench from version 7.5 and up. The input to the algorithm is mapped reads for ChIP-seq and genomic controls. The only parameter is a *p*-value threshold. We recommend a value of 0.05 for a more conservative peak-calling result, while a a more permissive value of 0.1 identifies also less pronounced peaks. The results of the algorithm are:

**QC Report** The QC report contains metrics about the quality of the ChIP-seq experiment. It lists the number of mapped reads, the normalised strand coefficient, and the relative strand correlation for each mapping. Furthermore, the QC report shows the mean read length, the inferred fragment length, and the window size used to model the signal shape. In case the input contains paired-end reads, the report will also contain the empirical fragment length distribution.

**Peak Shape Filter** the Hotelling Observer filter that was learned by the CLC shape-based peak caller.

**Peak Shape Score** The peak shape score value for every genomic position.

**Peaks** the list of all called peaks.

### Performance evaluation

There is a number of ChIP-seq peak calling packages available, each with slightly different implementation-dependent strengths and weaknesses. See for example the extensive comparison conducted by Wilbanks and Facciotti [36]. For clarity, in this paper we limit the comparative analysis of the CLC shape-based peak caller to three of the most popular state-of-the-art peak callers: the seqpeak tool included in the CisGenome software collection [12], the findPeaks tool included in the HOMER software collection [20] (http://homer.salk.edu/homer/ngs/index.html), and the MACS software [21, 40, 41]. These algorithms consistently rank among the top performing implementations [31] and will serve as reference points.

#### Gold-standard datasets

In this section, we present benchmark results from calling peaks in experiments targeted at identifying transcription factor binding sites (TFBS) from ChIP-seq data and at identifying accessible genomic regions from DNase-seq data.

ChIP-seq benchmark datasets often provide control data together with data from specific experiments (see [1] for guidelines on how to construct control samples). We will refer to the experiment samples as ChIP sample and to the control as control sample. In this paper, we use the data published by [31] as our gold-standard of truth. This dataset is based on expert curation of several hundred regions, each manually classified as either positive, negative, or ambiguous. The classification is done for three different ChIP-seq experiments, namely for the transcription factors MAX, NRSF, and SRF. In contrast to synthetic “spike-in” data generated by some authors, the use of manually annotated real-world data has the advantage of a blind experiment in the sense that the gold-standard classification is not produced by the very same persons developing the peak calling algorithm. The ChIP-seq datasets are:

**MAX** Published by Michael Snyder’s lab at Yale University and generated from cell-line K562 targeting *M*yc-*A*ssociated factor *X*.

**NRSF** Published by the Myers Lab at the HudsonAlpha Institute for Biotechnology and generated from cell-line Gm12878 targeting the *N*eural *R*estrictive *S*ilencer *F*actor (NRSF or REST) transcription factor.

**SRF** Published by the Myers Lab at the HudsonAlpha Institute for Biotechnology and generated from cell-line Gm12878 targeting the *S*erum *R*esponse *F*actor (SRF) transcription factor.

To show the generality of our method to similar sequencing data, we also investigate the performances of the peak callers on DNase-seq data. The signal produced by DNase-seq data is similar to the one produced by ChIP-seq data, so algorithms developed for ChIP-seq are commonly used to analyse DNase-seq data [42]. However, there are two main experimental protocols for DNase-seq, namely the “end-capture” [5] and the “double-hit” [4] protocols. Since the signal produced by the double-hit protocol [4] is very similar to the signal produced by a ChIP-seq experiment and peak callers are typically run with the same parameters used for the ChIP-seq data. On the other hand, the analysis of DNase-seq data obtained using the end-capture protocol requires specialised parameters in HOMER and MACS, as suggested in their user manuals (see Methods). Those parameter choices reflect the fact that the frame shift between reads mapping to the forward and reverse strand is not equal to the average fragment length as in ChIP-seq data, but is typically close to zero. We note that CisGenome was developed exclusively for analysing ChIP-seq data, so we used the default parameters for DNase-seq data. In this paper, we investigate two “end-capture” DNase-seq datasets:

**K562** Published by the Duke University and generated from cell-line K562 using the “end-capture” protocol.

**Gm12878** Published by the Duke University and generated from cell-line Gm12878 using the “end-capture” protocol.

The performances of the peak callers in these two datasets were computed using benchmark datasets of genomic regions independently validated using microarray data (see Methods).

Table 1 lists the amounts of reads in each dataset. We note that for MAX only one control replicate is available and that DNase-seq experiments do not have control data. The number of positive and negative regions in the ChIP-seq and DNase-seq gold-standard datasets are summarised in Table 2.

**Table 1:** Reads in benchmark datasets used

	MAX	NRSF	SRF	K562	Gm12878
Experiment Rep. 1	7,916,698	16,145,592	12,750,756	80,766,194	51,548,859
Experiment Rep. 2	5,947,320	26,619,271	12,291,355	138,607,720	12,252,754
Experiment Rep. 3				146,446,733	49,217,175
Experiment Rep. 4					64,324,983
Experiment Rep. 5					67,746,959
Control Rep. 1	13,510,465	16,377,339	14,164,649		
Control Rep. 2		14,363,052	16,222,442

**Table 2:** Number of positive and negative peaks in each of the gold-standard datasets

	MAX	NRSF	SRF	K562	Gm12878
#Positive peaks	225	138	134	927	712
#Negative peaks	62	66	46	927	712

#### Calculating performance metrics

**Fig. 11:**
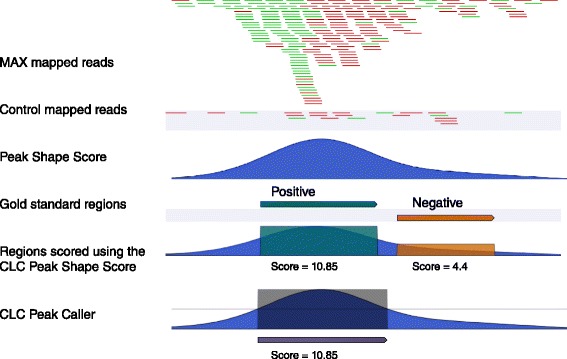
Assigning scores to validated regions. Two nearby regions, which are part of the gold standard, were annotated as positive (*cyan*) and negative (*orange*), respectively. Two methods for assigning scores are shown. 1) CLC Peak Caller: The result of the peak calling is a single peak (*purple bar*) overlapping the region validated positive and the score associated with it is the maximum score within the peak, i.e. 10.85. No peaks overlapping with the negative were found so no score will be assigned to the negative region. 2) CLC Peak Shape Score: The Peak Shape Score values within the positive and negative regions are extracted and the maximum values are used as score for the region. Note that this procedure always assigns a score to every region in the gold standard

The output from most peak callers is typically represented in the form of a ranked list of genomic sites, which are considered as peaks by the algorithm. This list of candidate peaks are ranked according to a statistical measure or score calculated by the algorithm, i.e. *p*-value, maximum coverage, fold enrichment, or a (e.g. log-transformed) combination thereof. Based on such ranked lists, the general framework for comparing prediction results of different predictors - with respect to a given gold-standard - is using Receiver-Operator Characteristic (ROC) curves, which plot the true positive over the false positive rate. For the CLC shape-based peak caller, we investigated both the performance of the peak caller and the performance of the Peak Shape Score. The main advantage of using the Peak Shape Score is that the result provides single nucleotide precision. Regions from the gold standard are scored as depicted in Fig. 11.

#### Running CLC shape-based peak caller with different input datasets

First, we investigated how the presence of a control experiment and the treatment of replicate experiments would affect the performance of the CLC shape-based peak caller. The analysis was performed using single replicates, using both replicates, and using a single file containing the reads of both replicates of each ChIP-seq datasets. The algorithm was then run without using control reads. In each analysis, the gold-standard regions were scored using the maximum of the Peak Shape Score in the region (see Fig. 11) and AUC-ROC (*A*rea *U*nder the *C*urve of the *R*eceiver *O*perator *C*haracteristic) values were estimated (Table 3).

**Table 3:** Area under the ROC curve values for different input settings

Experiment ChIP	Control ChIP	MAX	NRSF	SRF
Rep. 1 and Rep. 2	Rep. 1 and Rep. 2 ^a^	0.94	0.98	0.97
Merged	Merged ^a^	0.93	0.98	0.97
Rep. 1	Rep. 1	0.93	0.99	0.96
Rep. 2	Rep. 2 ^a^	0.95	0.97	0.97
Rep. 1 and Rep. 2	none	0.92	0.95	0.95
Merged	none	0.93	0.94	0.94
Rep. 1	none	0.90	0.95	0.94
Rep. 2	none	0.94	0.94	0.95

^a^For MAX the only available control replicate Rep. 1 was used as input (see Table 1)

The results in Table 3 show small differences between the performances obtained by using different choices of input, suggesting that the performances of the CLC shape-based peak caller do not degrade significantly when fewer data are available and even when no control data are available. As expected, running the algorithm using all available data consistently gave top performances, indicating that there is no need to perform pre- or post-processing steps when analysing datasets where replicates are available.

#### Results

We ran all peak callers (CLC shape-based peak caller, CisGenome, HOMER, and MACS) on the five datasets (Table 1). All tools were run initially with their respective default parameters, as this is how the software is used mostly. However, since all the peak callers by default do not output ambiguous peaks, many ROC curves would plateau and result in an unfairly low AUC-ROC value. Therefore, we relaxed parameters related to filtering of non-significant peaks, while leaving the other parameters unchanged. For CisGenome, we relaxed the cutoff for defining peak boundaries from 3 to 2.5; for HOMER, we relaxed the fold enrichment over input tag threshold from 4.0 to 2.0, the fold enrichment over local tag count threshold from 4.0 to 2.0, and removed the filtering step based on expected unique tag positions; for MACS we relaxed the q-value threshold from the default value of 0.05 to 0.25; and for the CLC shape-based peak caller, we relaxed the *p*-value threshold from 0.05 to 0.25 (see Methods). In all cases, the resulting AUC-ROC values obtained with these relaxed parameters were greater or equal to the AUC-ROC obtained using default parameters. We note that it was not feasible to remove the filtering steps altogether or to further relax the thresholds, because the resulting AUC-ROC values for CisGenome, MACS, and HOMER degraded in at least one dataset.

For CisGenome, we considered both the standard peaks (which we refer to as CisGenome) and the refined peak regions (which we refer to as CisGenome Refined), since the peak regions are typically very large. For MACS and HOMER, peaks were called after merging the reads from both replicates. For the CLC shape-based peak caller, both the Peak Shape Score and the results of the peak calling were collected as shown in Fig. 11. Then, for each dataset, an ordered list of peaks of decreasing confidence was obtained using the reported *p*-values of the peak regions. In this way, for each algorithm, the classified peaks are ordered according to the best scoring (lowest *p*-value) intersecting called peak or the worst *p*-value (1.0) if no called peak intersects. If ties exist in this ordering, peaks classified as negative are considered before peaks classified as positive. From this ordered list of positive and negative classified peaks, ROC curves and AUC-ROC values (Table 4) are produced. We discuss the results in the following section.

**Table 4:** Area under the ROC curve values

	MAX	NRSF	SRF	K562	Gm12878
CLC Peak Shape Score	0.94	0.98	0.97	0.98	0.97
CLC Peak Caller	0.91	0.98	0.95	0.98	0.96
CisGenome	0.82	0.94	0.96	0.87	0.87
CisGenome Refined	0.84	0.90	0.91	0.81	0.80
HOMER	0.85	0.96	0.93	0.92	0.92
MACS	0.92	0.96	0.97	0.96	0.95

**Fig. 12:**
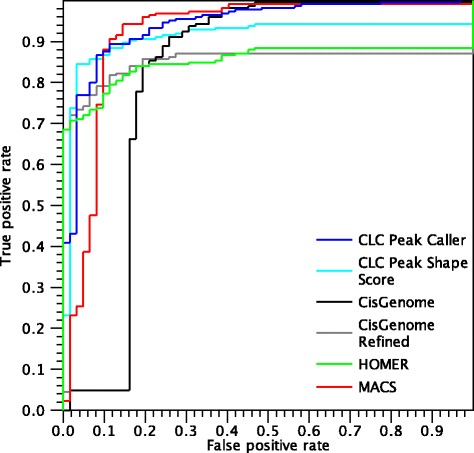
ROC *curves* for peaks found in the MAX dataset

The ROC curves for the experiments using the MAX dataset are shown in Fig. 12.

The first consideration is that HOMER makes its first mistake after reaching a true positive rate of 0.67, significantly later than the other algorithms. However, the performances of HOMER decline for more ambiguous peaks, resulting in a smaller AUC-ROC than MACS and the CLC shape-based peak caller. On the other hand, MACS makes more mistakes in the beginning, but it is able to identify nearly all peaks, resulting in a higher AUC-ROC value. The CLC shape-based peak caller behaves similarly to HOMER at low false positive rate but is able to call more peaks. We note that in this dataset there is a difference in the ROC curves of the CLC Peak Shape Score and the CLC shape-based peak caller. This is due to the fact that many regions of the gold standard are situated near the centre of a peak. Therefore, in this context, the performance value depends significantly on the ability to identify the correct peak boundaries. For example, a negative region close to a peak region is shown in Fig. 11. In this example, the peak caller correctly identifies that there is no peak in the negative region, while the CLC Peak Shape Score approach assigns a moderate score to the negative region because it is in proximity of a peak. Conversely, a situation where a positive region is situated near a strong peak may be missed by the peak caller, which calls only the main peak and may not extend the boundaries enough. This makes the two ROC curves different, making the peak caller more appropriate for small false positive rates, whereas the Peak Shape Score approach assigns a score to every genomic position. The difference between CisGenome and CisGenome Refined is even more pronounced, as the peaks called by the default CisGenome are too large and often include nearby regions annotated as negatives sites. On the other hand, using refined peak boundaries drastically improves the performances of the algorithm for this dataset, although it misses some peaks of lower quality.

**Fig. 13:**
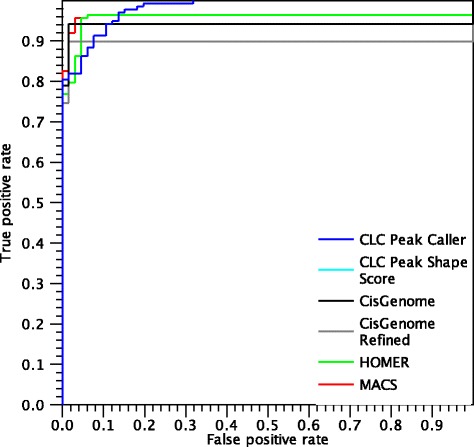
ROC *curves* for peaks found in the NRSF dataset

Figure 13 shows the ROC curves for called peaks in the NRSF dataset.

The performances of all the algorithms are very good in this dataset and all algorithms make their first mistake after a true positive rate of 0.7. The main difference between the CLC shape-based peak caller and the other two algorithms is that, although it makes a few mistakes earlier, the peak caller is able to call nearly all peaks, resulting in a higher overall AUC-ROC value. In this dataset, the performances of the CLC shape-based peak caller and the Peak Shape Score are equal, because there is no ambiguity regarding peak boundaries. Therefore, the two ROC curves completely coincide and only the curve from CLC Peak Caller (blue) is visible. The performances of the two CisGenome variants are quite similar, but CisGenome Refined misses some peaks that the default CisGenome correctly identifies.

**Fig. 14:**
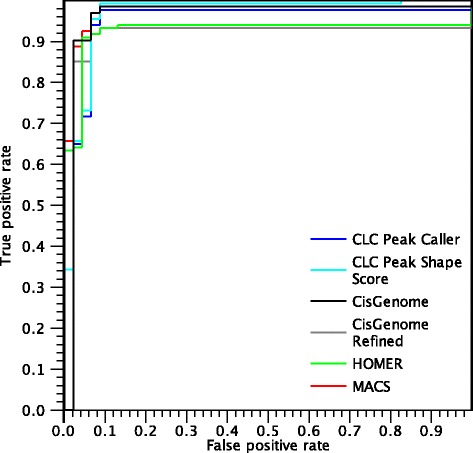
ROC *curves* for peaks found in the SRF dataset

Figure 14 shows the ROC curves for called peaks in the SRF dataset.

Similarly to the NRSF dataset, most positive peaks are called by all algorithms and only a few mistakes are made, resulting in good performances for all the algorithms. MACS performs better than the other algorithms for small false positive rates, resulting in the highest AUC-ROC value. In this dataset, the difference between the peak caller and the Peak Shape Score is very small and the curves are very close to each other. The main difference is that the peak caller does not identify all peaks and plateaus slightly before reaching the top of the plot. On the other hand, the Peak Shape Score gives a score to every region in the gold standard, so it is always able to reach a true positive rate of 1.

**Fig. 15:**
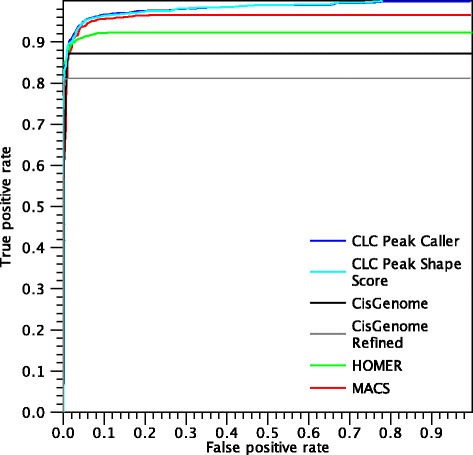
ROC *curves* for peaks found in the K562 dataset

**Fig. 16:**
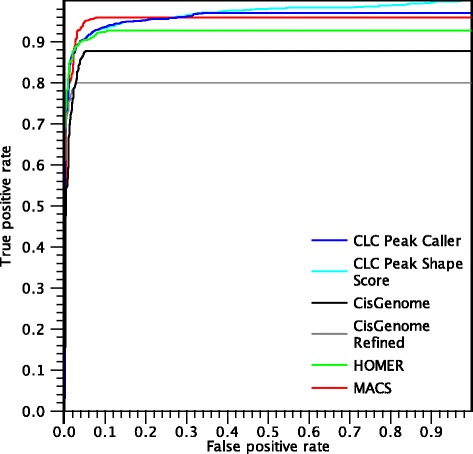
ROC *curves* for peaks found in the GM12878 dataset

Figures 15 and 16 shows the ROC curves for called peaks in the DNase-seq datasets.

The ROC curves look similar to the ones obtained for the ChIP-seq data, in particular we note that the AUC-ROC values are high and that all algorithms are able to identify at least 80 % of the positive peaks making only a few mistakes.

Table 4 summarises the results by the area under the ROC-curve values. MACS and the CLC shape-based peak caller have the highest values, while HOMER is often penalised because it misses several peaks and CisGenome does not often identify the correct peak boundaries. Similarly to the MAX dataset, in the GM12878 dataset, MACS performs better than the other algorithms for false positive rates between 0.1 and 0.3, while the CLC shape-based peak caller performs marginally better with low false positive rates and is able to identify more positives and thus results in a higher overall AUC-ROC. On the other hand, the ROC curve of CLC shape-based peak caller in the K562 dataset shows better performances than the other algorithms consistently for all false positive rates.

Even though it is hard to directly compare the results of the Peak Shape Score with more traditional peak calling algorithms, we observe that there are clear advantages in having a score with single nucleotide resolution, especially for calling low-quality peaks. The results for the DNase-seq show similar trends to the results of the ChIP-seq analysis.

### Broad peak ChIP-seq data

To further show the generality of the approach, we next applied the methodology to histone mark ChIP-seq data, which present broad peaks with variable length. For classifying these broad-peak patterns, we adapted the peak-shape approach to use annotated gene regions as additional input. Rather than considering coverage shapes in a fixed-sized window around each genomic position, shapes in predefined input regions of variable length are computed by scaling to a unit-window.

The analysis was performed using the same learning procedure, with only few differences. First, genomic coverage was normalised in the same way as the normal algorithm. Instead of considering positive and negative reads as separate inputs, the normalised read coverages were aligned and summed as *g*^′^(*x*)=*f*^′^(*x*+*μ*/2)+*r*^′^(*x*−*μ*/2), where *f*^′^(*x*) is the normalised coverage of reads mapping to the forward strand, *r*^′^(*x*) is the normalised coverage of reads mapping in the reverse strand and *μ* is the fragment length mean. This step was necessary since the displacement between forward and reverse reads is a global property of the experiment and does not depend on the variable gene length. The shapes of all genes transcribed in the reverse strand were reversed.

The initial discovery of obvious peaks was done similarly to normal algorithm, the only difference being that instead of computing the Peak Shape Score for each genomic position by deconvolution (Eq. 1), we calculated a single score for each gene region by computing the dot product of the reshaped profile and the filter. The shape learning phase and *p*-value computation were done identically to the standard algorithm, returning a score and a *p*-value for each gene.

We then assessed the quality of the prediction by checking whether we could predict gene expression from ChIP-seq data of histone modification marks known to be associated with expression [17, 43, 44]. ChIP-seq data for the histone modifications H3K36me3, H3K4me3, H3K9ac, H3K27ac for the four human cell lines GM12878, HepG2, Huvec and Nhek [43]. We then derived a gold standard of expressed and non-expressed genes from RNA-seq data [45], which we used to compute AUC-ROC values (Table 5) as described in the Methods section.

**Table 5:** Area under the ROC curve values for histone mark ChIP-seq data

	GM12878	HepG2	Huvec	Nhek
H3K36me3	0.96	0.95	0.95	0.95
H3K4me3	0.89	0.89	0.89	0.89
H3K9ac	0.92	0.91	0.91	0.92
H3K27ac	0.90	0.93	0.91	0.91

The performances of the method are consistent among the four cell lines, indicating that the method is robust. We note that the difference in predictive performance between the different datasets is most likely due to the fact that some histone marks (e.g. H3K36me3 [46]) correlate more strongly with active transcription than the other marks.

## Conclusions

In this paper, we discussed the state-of-the-art and current trends in the field of peak-detection algorithms that lead to the development of the new CLC shape-based peak caller. We described our implementation of a general and statistically well founded algorithmic engine, applicable to a wide range of different datasets. This flexibility is combined with specificity by automatically learning the characteristics of the signal present in the data.

### Discussion

The systematic performance evaluation of the CLC shape-based peak caller on a published manually curated reference dataset shows that it compares favourably to popular current algorithms. It is important to note that the existing peak callers are readily tuned to detect narrow peaks at transcription factor binding-sites and hence are very well suited to the benchmark data. Hence, it is reassuring to see the new CLC shape-based peak caller performs on the same level and sometimes even better than CisGenome, HOMER, and MACS.

Besides the theoretical advantages of the peak-shape approach and the positive results obtained on the benchmark data, in practice it is equally important that the CLC shape-based peak caller wraps the underlying algorithm and statistics into an automated pipeline, which is simple to use for non-specialists. At the same time, we provide the option of further optimisation by manually delineating positive and negative examples - a process that is much easier understood and visualised than abstract parameters. Moreover, it is of great importance to make sure that the input data has sufficient quality and consequently that the results can be trusted. Therefore, potential problems are highlighted by detailed quality reports.

To our knowledge, the CLC shape-based peak caller is unique in its combination of flexibility and accuracy through the ability of building optimised filters for different analysis tasks and datasets. This enables sustained development and maintenance of the tool set in exploratory research, since the adaptation to different filters does not require changes to the underlying code base. At the same time, existing filters are transferable and can be applied to new datasets as they become available in production settings, so there is no need to re-learn and optimise the filters from scratch if the same peak-shape is to be detected.

### Future developments

The CLC shape-based peak caller represents an important building block within a larger ecosystem of NGS data analysis software. Hence considerations for further developments have to take into account the interplay with other related components. From a user centric view, major improvements will be gained by seamless interoperability with both upstream and downstream analysis steps. Since peak detection is now handled by a flexible and generally applicable algorithmic engine, the focus shifts from algorithmic issues to data integration, downstream analyses, and visualisation capabilities.

The typical analysis downstream of peak finding or classification are motif finding for Transcription-factor ChIP-seq data, the annotation of peaks with nearby genes for functional analysis such as gene set enrichment and the correlation with other omics datasets such as gene expression data.

A great deal of interoperability is already taken care of by the integration of the CLC shape-based peak caller into the track-based framework of CLC Genomics Workbench. By taking its inputs from tracks and producing outputs in the same format, it can be freely combined with other track-based tools. Therefore it is easily integrated into larger analysis pipelines and workflows while benefiting from the ongoing improvements to the visualisations and operations available for track-based data.

A major advantage of the CLC shape-based peak caller is that it is not exclusively designed to detect signals in transcription factor ChIP-seq datasets. Herein, we have shown that it could be directly applied to the DNase-seq data without having to change parameters and still yielding comparable performances. In principle, the methodology is applicable to detect signals in data from a wide range of different sequencing protocols, including FAIRE-seq, broad-peaks from ChIP-seq of histone-modifications or other epigenetic marks such as DNA methylation.

## Methods

### ChIP-seq data

The manually curated peak annotation by [31] are obtained via http://tare.medisin.ntnu.no/chipseqbenchmark/downloads/ChIPSeq_files_in_bed_format/.

The corresponding original data from ENCODE (release 1) are available via the following links: http://hgdownload.cse.ucsc.edu/goldenPath/hg19/encodeDCC/wgEncodeSydhTfbs/ and http://hgdownload-test.cse.ucsc.edu/goldenPath/hg18/encodeDCC/wgEncodeHudsonalphaChipSeq/release1/.

### DNase-seq data

End-capture [5] DNase-seq data for the cell lines K562 and GM12878 used in this study were produced by the ENCODE consortium [45]. The mapped.bam files “end-capture” [5] DNase-seq and validations were obtained from http://hgdownload.cse.ucsc.edu/goldenPath/hg19/encodeDCC/wgEncodeOpenChromDnase/.

Validation was performed by simultaneously generating microarray-based data from the same material used for DNase-seq. The microarray used was a Nimblegen tiling array that covers the 1 % of the genome that was the focus of the pilot encode project.

### Peak callers

CisGenome version 2.0 was downloaded from http://www.biostat.jhsph.edu/~hji/cisgenome/index_files/download.htm. The seqpeak tool was run with parameters “-c 2.5”. The columns “start” and “end” were chosen to define the peak regions and the columns “left_peakboundary” and “right_peakboundary” were chosen to define the refined boundaries. In both cases, the “rank” column was used to build the ROC curve.

HOMER version 4.7 was downloaded from http://homer.salk.edu/homer/download.html. The findPeaks tool was run with parameters “-style factor -F 2 -L 2 -C 0” for ChIP-seq and with the parameters “-style dnase -F 2 -L 2 -C 0” for the “end-capture” DNase-seq data. The column “findPeaks Score” was used to build the ROC curve. We note that removing the filtering options -F -L -C resulted in dramatically reduced performances, so the threshold for -F and -L was relaxed, instead.

MACS version 2.1.0 was obtained from https://github.com/taoliu/MACS/. The software was run with the parameter “–qvalue 0.25” for ChIP-seq and with the parameters “–qvalue 0.25 –nomodel –shift -100 –extsize 200” for the “end-capture” DNase-seq data The column “q-value” was used to build the ROC curve.

The CLC shape-based peak caller version 1.0 was run with the parameter “–*p*-value 0.25”. The Score Regions tools from Advanced Peak Shape Tools Plugin version 1.0 beta 3 was used to assign Peak Shape Scores to gold standard regions.

### Broad peak ChIP-seq data

Histone modification ChIP-seq data for four cell lines GM12878, HepG2, Huvec and Nhek were collected from the Sequence Read Archive. The human genome hg19-GRCH37 was used as reference. Paired-end RNA-seq data from Encode/Caltech [45] were downloaded from SRA (GM12878: SRX159821, HepG2: SRX159823, Huvec: SRX159825 and Nhek: SRX159827) and analysed using the RNA-seq Analysis tool of the CLC Genomics Workbench 8.0 the using the parameters “Count paired reads as two = Yes”, “Expression value = RPKM” and “Calculate RPKM for genes without transcripts = Yes”. Overlapping genes were merged and the resulting region was counted once. A gene was considered to be expressed if its RPKM was >2 in all RNA-seq replicates and a gene to not be expressed if its RPKM was <2 in all replicates. This procedure resulted in 6478, 6311, 7025 and 6136 positive regions and 17346, 17894, 17786 and 17583 negative regions for GM12878, HepG2, Huvec and Nhek, respectively.

ChIP-seq data for the four cell lines [43] was downloaded from SRA (study ID: SRP005344) and was mapped to the human genome using the Map Reads to Reference tool of the CLC Genomics Workbench 8.0 using the option “Non-specific match handling = Ignore”. For each histone modification mark, all ChIP-seq replicates from the same cell line were used as input to the peak-shape analysis. The resulting Peak Shape Score was used to compute AUC-ROC values.
